# The quality of life and its inter-relationship with posttraumatic stress disorder and social support in two post-conflict communities in Nigeria

**DOI:** 10.1177/13591053231222851

**Published:** 2024-01-22

**Authors:** Oluyemi O Akanni, Aladi N Edeh, Michael T Agbir, Anthony A Olashore

**Affiliations:** 1 Federal Neuro Psychiatric Hospital, Nigeria; 2 Benue State University, Nigeria; 3 University of Botswana, Botswana

**Keywords:** conflict, PTSD, quality of life, social support

## Abstract

The study aimed to compare the quality of life (QoL) in two communities with different exposures to conflict and investigate the inter-relationship between posttraumatic stress disorder (PTSD), social support, and QoL. This is a cross-sectional with 413 participants. Study instruments included the PTSD module of the Composite International Diagnostic Interview (CIDI), the World Health Organization Quality of Life BREF (WHOQoL-BREF), and the Multidimensional Scale of Perceived Social Support (MSPSS). The family domain of social support was protective of both PTSD and QoL. Except for the relationship between community’s location and the physical subscale of the QoL, a hierarchical regression analysis showed that all the independent variables were significantly associated with the QoL domains. Direct exposure to crises impaired QoL more than areas indirectly exposed. PTSD and the family domain of social support play a significant role in the QoL outcome. This suggests that therapeutic intervention to improve QoL should target these key variables.

## Introduction

The World Health Organization (WHO) defines quality of life (QoL) as individuals’ perception of their position in life in the context of the culture and value systems in which they live concerning their goals, expectations, standards, and concerns. It can be considered as the individual’s current happiness or pleasure, as a subjective rating by a person of their level of functioning in various aspects, and objectively as an assessment of whether certain health objectives have been met (WHO, 2012).

Quality of life assessment is a multidimensional construct that examines several aspects of functioning. The WHO, in its assessment of the quality of life, relates it to the physical health, psychological state, social relationships, and the environment of the person as well as their general health and general quality of life (WHO, 2012). Although there are criticisms that areas of assessment reflect the clinician or researcher’s preference rather than areas of priority for the patient, quality of life can measure health outcomes, help the clinician to know areas in an individual’s life requiring intervention, as well as assist in appraisal of healthcare (Pelcovitz et al., 1996). For instance, optimizing outcomes in functioning is now considered the goal of managing people with mental disorders and not only treating their symptoms because quality of life enables clinicians to determine the impact of the illness (Athanasiou et al., 2023). Furthermore, quality of life is fundamental to other aspects such as resource allocation, research outcomes, care satisfaction, support services quality, and healthcare options (Rapkin and Schwartz, 2004). Consequently, interest in quality of life is progressively gaining significance in medical practice and research.

Posttraumatic stress disorder (PTSD) can affect the quality of life of individuals in different areas of functioning. PTSD is defined as an abnormal reaction suffered from exposure to a traumatic event that the individual was a victim of, witnessed, was confronted with, or learned about a loved one. It is associated with intrusive memories or unpleasant dreams of the event, avoidance of memories or reminders of events, and symptoms of hyperarousal, which should have been present for about a month (DSM-5). Several studies have found a lower quality of life in persons with PTSD than their counterparts without PTSD (Ayanda et al., 2014; Braš et al., 2011; Neethu and AbdulRafeeque, 2015). For example, Neethu and Abdul conducted a study in India’s Pondicherry area after an underwater tsunami caused an earthquake, leading to destruction. They examined the impact of PTSD on the quality of life of 260 subjects comprising 130 tsunami victims and 130 unaffected people aged between 25 and 40 years using the World Health Organization Quality of Life-BREF (WHOQoL BREF). The study found that people with PTSD had a lower quality of life than those without PTSD in all assessment domains (Neethu and AbdulRafeeque, 2015).

The reason why individuals diagnosed with PTSD have a lower quality of life when compared to those without PTSD may be as a result of the former experiencing impairment more in their functioning (Glassman et al., 2019) because the disorder can be disabling. However, research indicates that the severity of the traumatic events experienced, the intensity of the trauma, the extent of dysfunction of the central nervous system, and the availability of post-conflict health and social support services can all play a role in determining the level of psychological distress and impairment experienced by a person with PTSD (Bravo-Mehmedbasić et al., 2010).

Besides psychological measures, social support constitutes another variable that influences the quality of life of people (Kumcağız and Şahin, 2017; Şahin et al., 2019; Zdun-Ryżewska et al., 2018; Zhang et al., 2021). Social support is a multidimensional construct, and it is the help one gets through interactions with others (Kaniasty, 2005). This help can be from friends, family, neighbors, colleagues, partners, or others. It can come in the form of emotional support (empathy), tangible support (practical help), or informational support (advice) (Kaniasty, 2005). Literature reveals that it consists of the actual size of help (amount of support), the perception of that assistance as helpful, and the degree to which the individual uses the available support (Fleury et al., 2009; Kaniasty, 2005; Klarić et al., 2008). Therefore, it is suggested that individuals benefit more from their perception of social support as beneficial than the amount of support they receive (Klarić et al., 2008).

Research has majorly suggested that the way through which social support works to reduce a negative psychological outcome is the stress buffer and main effect methods (Helgeson, 2003). The stress-buffering model suggests that social support protects people from the harmful effects of stress, while the main effect model talks about interaction in a social network, which helps create a sense of belonging love and helps one pick a psychologically beneficial lifestyle (Beiser et al., 2010).

Many studies have reported that perceived social support from family, friends, and co-workers reduces the occurrence of PTSD (Beiser et al., 2010; Klarić et al., 2008). In a study of PTSD and social support, Klaric et al. investigated the correlation between social support and PTSD symptoms in women aged between 28 and 65 who had experienced war in Herzegovina (Klarić et al., 2008). The participants, selected by systematic random sampling, consisted of an experimental group of 187 women in Mostar who had experienced war, while the control group had 180 women in the area around Mostar who had been exposed to the news of the war. PTSD was present in 53 (28.8%) of the women in the experimental group and eight (4.4%) in the control group. Women in the experimental group with PTSD had significantly lower levels of perceived social support from friends (*t* = 2.91; *p* < 0.05) and co-workers (*t* = 2.30; *p* < 0.05). Nevertheless, the study did not evaluate quality of life and its variation in the two groups.

Moreover, social support is related to a phenomenon called posttraumatic growth, which is understood as the occurrence of positive psychological change that can come about when individuals respond to a highly challenging life (Swickert and Hittner, 2009). However, some studies have found no relationship between PTSD and social support, suggesting the possibility of other factors mediating this relationship (Clapp and Gayle Beck, 2009; Nwoga et al., 2016). For instance, negative attitudes and interactions within social networks can cause poor psychological outcomes (Clapp and Gayle Beck, 2009). It is not yet clear from available empirical evidence whether the level of exposure to conflict can confound the relationship between social support and PTSD. However, what has been found is that social support can attenuate the link between torture exposure and PTSD (Gottvall et al., 2019).

Social support has also been linked to quality of life. In an in-depth interview study conducted by Yao and his colleagues among 349 stigmatized patients who sought online support, they categorized support into emotional support, informational support, companionship and relatedness, and grouped quality of life into physical, psychological and existential domains. They found among the categories of support that the impact of emotional support was the most effective on the psychological quality of life, though other dimensions of support affected other domains of quality of life. What was influential in this outcome was the patient’s perception of their level of social exclusion: when they perceive high levels of social exclusion, they seek a variety of online social support and attain a more improved quality of life than those patients with lower levels of social exclusion. Though perception played a key role in the link between the two, other researchers have explained the ability of social support to build resilience and promote positive attitudes to improve quality of life (Sippel et al., 2015).

It is with this background that the study aimed to compare the quality of life in two communities in Nigeria that have experienced different levels of conflict and to explore the relationship between quality of life, posttraumatic stress disorder (PTSD), and social support. Nigeria, a country with diverse ethnic groups, has witnessed several ethnic conflicts. One such conflict is the Tiv-Fulani herdsmen crisis in Benue State, which occurred between 2013 and 2014 and affected some of the state’s local governments, including the Guma Local Government Area (Dura, 2014). In the affected community, some people lost their lives in the crisis, others were maimed, and others experienced the destruction of their properties. Since, to the best of our knowledge, studies on quality of life in the context of this crisis in Guma local government are lacking, we embarked on the study.

We constructed a conceptual framework that the location of the community (in terms of direct or indirect exposure to conflict), social support and PTSD will directly influence the quality of life. Further, we hypothesized that the quality of life would be poorer in the community with direct exposure to crises than those with indirect exposure because the former will suffer greater psychological and physical ill-health and environmental damages. Moreover, since Posttraumatic Stress Disorder (PTSD) is a frequent psychological aftermath of conflict whose rate varies with exposure severity (Musisi, 2004), we posited that PSTD will negatively impact the quality of life. Theoretically, quality of life has been explained in terms of the hierarchical needs of human satisfaction. A society with higher-order needs (such as social, esteem, and self-actualization) will have a higher quality of life when compared with communities with lower-order needs (biological and safety-related needs) (Sirgy, 1986). This theoretical model, drawn from the Maslov developmental perspective, supports the conceptual framework of people living in areas directly devasted by the crisis reporting a lower quality of life compared to those not directly affected because of higher safety and medical concerns. Finally, we hypothesized that social support would be protective of (inversely related to) quality of life and further serve as a mediator on PTSD.

## Methods

### Study location and population

The study occurred in Uikpiam and Daudu, two communities with similar socioeconomic activities and culture, in Guma Local Government Area (LGA) of Benue state, North Central Nigeria. Guma is located in the North-Eastern part of Benue State with an area of 2882 km^2^ and a population of 194,164 as of the 2006 census (NPC, 2006). Makurdi, Tarka and Logo LGAs bind the Local Government. Most of the inhabitants are farmers, with fishing as the second-largest economic activity in the Local Government. About 95% of the population is Tiv in ethnicity, and most people in Guma understand and speak the Tiv language. Guma LGA comprises 10 council wards, with its headquarters in Gbajimba, some of which were affected by the 2013–2014 Tiv-Fulani crises.

The 2013/2014 crisis between Fulani herdsmen and Tiv farmers in the Uikpiam community in the Mbabai council ward resulted from a misunderstanding, leading to attacks and counterattacks from both sides. During this dispute, houses were burnt and destroyed, many persons were injured, and others were killed. Inhabitants of the Daudu community in the Mbawa council ward, 8 km away from Uikpiam, indirectly experienced the impact of the crises in some ways. Due to the crisis, a camp was temporarily set up in Daudu to house displaced persons from Uikpiam. Also, there was a heavy presence of security personnel to guard the camp to prevent an escalation of the crisis to this place. Moreover, the inhabitants of Daudu were regaled with tales of individuals’ trauma experiences, including those of loved ones.

### Selection criteria

The inclusion criteria were residents of Uikpiam and Daudu aged between 18 and 65 years and people who were physically present in the village at the time of crisis. Eligible potential participants with pre-existing psychopathology or conspicuous severe cognitive impairment that made the interview practically impossible for them were excluded. The presence of pre-existing psychopathology was ruled out using a yes or no answer to a question that simply asked if the person had ever had any history of mental illness in the past or present, excluding PTSD.

### Sample size determination

The study was conducted among a sample of the two selected communities. Therefore, the only available population sizes for the communities of the study were in the gazette for 1991, which were 743 persons for Uikpiam and 1531 persons for Daudu (NPC, 2006). However, at a projected growth rate of 3%, the population extrapolation estimates for Uikpiam and Daudu were 1722 and 3548, respectively (Christopher, 2003).

Furthermore, using the Vaughan formula (Vaughan and Morrow, 1989) to obtain the minimum sample size where *Z* is set at a 5% significant level = 1.96 and *p* is 41%, which is the estimate from a previous study of the prevalence of PSTD among post-conflict communities in Jos, Nigeria (Obilom and Thacher, 2008); a sample size of 372 was calculated. To account for a 10% non-response rate, we determined that a minimum sample size of 413 was necessary. Using proportions based on Uikpiam and Daudu’s projected population, we calculated separate sample sizes of 135 and 278, respectively.

### Sampling technique

The study was cross-sectional, and a multistage cluster random sampling method was used. The multiple stages included the Local Government level, followed by the council ward, then enumeration areas (EA), and the level of the houses. At the local government level, out of the six local government areas affected by the Fulani/Tiv crisis of 2013/2014, Guma was chosen by a simple random balloting method. Guma is, however, divided into 10 council wards.

At the level of the council wards, there was a stratification of the wards into those directly affected by conflict and those not directly affected by conflict. Through a simple random method, one community each was selected from those directly affected (Uikpiam) and those not directly affected (Daudu). Nonetheless, each community is divided into EAs by the National Population Commission. At the level of EAs, Uikpiam is divided into seven, while Daudu is divided into 20 EAs. By the simple random method, six out of the seven EAs in Uikpiam and six out of the 20 in Daudu were selected. The sample population needed in each community was distributed among the six EAs in each community.

In Uikpiam, the 135 persons were divided by the six EAs to obtain 22 persons in three EAs and 23 in the remaining three EAs; while in Daudu, the 278 persons were distributed among the six EAs to get 46 persons per EA except for the sixth EA in which 48 persons were selected. The maps of the EAs used were updated with the help of some youths in the community before the interview. With the help of the maps, the numbers of houses in each EA were available.

The number of houses was divided by the number of people needed in each EA to determine the house interval. Then, in each house, all the persons who met the eligibility criteria were interviewed with the questionnaires.

### Study instruments

1. Questions on socio-demographic variables such as age, gender, marital status, educational level, and occupational status.
2. Quality of Life: This was measured using the World Health Organization Quality of Life-BREF (WHOQoL-BREF, 1998, a shorter version of the World Health Organization Quality of Life questionnaire. It includes four main domains: physical health, psychological health, social relationships, and environment, and contains 26 questions that were measured on a 5-point Likert scale. It is more convenient for use in large research studies or clinical trials because of the brevity. Items are rated on a 5-point Likert scale (a low score of 1 to a high score of 5), with a higher score indicating a higher quality of life. It has been shown to have satisfactory discriminant validity, internal consistency, and test-retest reliability (Ryan, 2021). This instrument has been used extensively in various studies comparing the quality of life in communities, including Nigeria, among refugees and non-refugees (Akinyemi et al., 2012).^24^ A satisfactory Cronbach alpha of 0.84 was recorded in this study.
3. The PTSD module of the Composite International Diagnostic Interview (CIDI) (WHO, 1997): The section of the PTSD module of CIDI was used for this study. The CIDI is a fully structured interview that makes diagnoses according to ICD 10 and DSM IV. The inter-rater reliability is excellent, while the test-retest reliability and validity are good (Andrews and Peters, 1998). It has been used to study PTSD in Nigeria (Beiser et al., 2010; Gureje et al., 2006), and a marginally acceptable Cronbach alpha of 0.60 was measured in the present study.
4. Multidimensional Scale of Perceived Social Support (MSPSS) (Dahlem et al., 1991): This scale was developed by Zimet and colleagues using adult samples. It has been used to measure perceived social support in different cultures (Dahlem et al., 1991) and is low in social desirability bias (King et al., 1995). It has 12 items that determine family support, friends support, and significant others support. It uses a Likert scale from 1 “very strongly disagree” to 7 “very strongly agree.” Items 3, 4, 8, and 11 measure family support; items 6, 7, 9, and 12 measure friend support, while items 1, 2, 5, and 10 measure significant other support. It has both subscale scores and total mean scores. A higher score indicates higher support. It has high internal consistency with Cronbach alpha of 0.86 (Shalev et al., 2006). It has been validated for use in Nigeria (Folasire et al., 2014; Mohammad et al., 2015), and a high Cronbach alpha of 0.90 was measured in this study.

### Ethical consideration

Ethical approval to conduct the study was obtained from the Institutional Review Board. Additional permission was obtained from the Local Government Headquarters, and informed consent from the participants. Participation was voluntary, and no harm came to participants because of the study. Those with clinically significant PTSD were referred to a mental health facility for treatment.

### Study procedure

All the instruments used were translated into the Tiv language. A back-to-back iteration translation was done by professionals such as clinical psychologists and psychiatric nurses who have good knowledge of the subject matter and are well-literate in Tiv and English to ensure the instruments remain unaltered. After translation, it was then pre-tested on 10 participants selected from one of the EAs in Daudu not to be used in the study.

The interview was conducted with the help of four trained mental health professionals who spoke both the Tiv and English languages fluently. In addition, they were adequately trained to use the instruments by one of the authors (AMT). The main study took place between September and October 2018, while a pilot study was carried out a week before the main study. Ten percent of the participants not used in the main study were used for the pilot study (Connelly, 2008). Observations made during the study were useful in modifying the study procedure.

Before the interview, the research assistants screened subjects to determine eligibility by asking for ongoing or previous behavioral and emotional problems, apart from PTSD, that caused them distress or interfered with functioning.

### Data analysis

The data collected was cleaned and analyzed using the statistical package for social sciences IBM version 23. The socio-demographics in Uikpiam and Daudu were calculated in percentages, of which a comparison between the two communities was made using a Chi-Square (χ^2^) test. The independent *t*-test was computed to determine differences in the mean scores of the various quality of life between the two communities. Pearson correlation was used to determine correlations between the sub-scales of quality of life, social support components, and PTSD. Moreover, a two-step hierarchical multiple regression was carried out. The community’s location was first entered to control its effect on the quality of life. Thereafter, the remaining significant variables, such as PTSD and family social support, were entered in Step 2 to determine their independent effects on quality of life. The level of significance was set below 0.05.

## Results

### Socio-demographic profile of participants

Table 1 shows the distribution of the participants by socio-demographic profile. A total of 413 participants were interviewed, comprising 135 subjects from Uikpiam and 278 from Daudu.

**Table 1.** Socio-demographic characteristics of participants.

Variables	Community of residence				
	Uikpiam (*N* = 135)	Daudu (*N* = 278)	Total (*N* = 413)	χ^2^	*p*-Value
	Frequency (%)	Frequency (%)	Frequency (%)		
Sex					
Male	59 (43.7)	166 (59.7)	225 (54.5)	9.39	**0.00**
Female	76 (56.9)	112 (40.3)	188 (45.5)		
Age					
Young	89 (65.9)	201 (72.3)	290 (70.2)	5.13	0.08
Middle	35 (25.9)	68 (24.5)	103 (24.9)		
Old	11 (8.1)	9 (3.2)	20 (4.8)		
Marital status					
Single	26 (19.3)	57 (20.5)	83 (20.1)	4.09	0.13
Married	95 (70.4)	207 (74.5)	302 (73.1)		
Others	14 (10.4)	14 (5.0)	28 (6.8)		
Education					
None	49 (36.3)	82 (29.5)	131 (31.7)	7.96	0.05
Primary	48 (35.6)	79 (28.4)	127 (30.8)		
Secondary	31 (23.0)	101 (36.3)	132 (32.0)		
Tertiary	7 (5.2)	16 (5.8)	23 (5.5)		
Occupation					
Farmer	113 (83.7)	199 (71.6)	312 (75.5)	7.23	**0.01**
Non Farmer	22 (16.3)	79 (28.4)	101 (24.5)		

Significant *p*-value in bold.

About half (54.5%) of all participants were males, with the majority (73.1%) married. Those with no formal education were 31.7%, while altogether, 68.3% had some form of education, including primary, secondary, and tertiary education. Over three-quarters are farmers, and 24.5% are not farmers. Furthermore, the participants from the two communities differed only in their gender and occupation. The participants from Uikpiam had more females and farmers than the other community.

Table 2 shows that the participants from Uikpiam significantly had lower scores on all the quality of life subscales than those from Dandu.

**Table 2.** Location differences in mean scores of the quality of life variables.

Variables	Uikpiam	Dandu	*t*	*p*
Physical	11.94	12.51	−2.61	**0.010**
Psychological	13.11	14.26	−5.95	**0.00**
Social relationship	14.12	15.34	−3.82	**0.00**
Environmental	12.74	14.60	−5.75	**0.00**

Significant *p*-value in bold.

PTSD negatively correlated with all four subscales of the quality of life, with the correlation coefficient (r) ranging from −0.212 to −.425. However, PTSD did not correlate significantly with any of the social support subscales except for the negative correlation with the family component (*r* = −0.200). Furthermore, only the family subscale of social support consistently correlated positively with all the subscales of the quality of life. The inter-correlations of the remaining social support (friends and significant others) with quality of life subscales were inconsistent, weak, and positive (Table 3).

**Table 3.** Inter-correlation of social support PTSD, quality of life variables.

Measures	1	2	3	4	5	6	7	8
1. Physical	1							
2. Psychological	0.405**	1						
3. Social relationship	0.415**	0.467**	1					
4. Environmental	0.431**	0.615**	0.516**	1				
5. Friend	0.147**	0.017	0.201**	0.053	1			
6. Significant others	0.019	0.006	0.154**	0.072	0.383**	1		
7. Family	0.155**	0.241**	0.265**	0.305**	0.315**	0.472**	1	
8. PTSD	−0.212**	−0.318**	−0.234**	−0.425**	0.066	0.067	−0.200**	1

** Correlation is significant at the 0.01 level (two-tailed).

* Correlation is significant at the 0.05 level (two-tailed).

Hierarchical multiple regression was used to assess the effect of PTSD and the family social support on all the quality of life subscales after controlling for the influence of the community. The community’s location was entered in Step 1 and found to explain between 2.2% and 8.3% of the variance for the various quality of life. After the entry of PTSD and the family social support in Step 2, the total variance explained by the model somewhat increased. The findings were quite remarkable, with a significant increase observed in all groups, ranging from 4.2% to 17%, at a *p*-value <0.001. In the final model, all the dependent variables were statistically significant, except for community’s location in the physical subscale of the quality of life (*B* = 0.082, *p* = 0.105) (Table 4).

**Table 4.** Hierarchical regression analysis for location, family social support and PTSD predicting quality of life subscales (*N* = 413).

		Model 1			Model 2		
QoL subscales	Predictors	β	*p*-Value		β	*p*-Value	
Physical	Location	0.148	0.003	*R*^2^ Δ = 0.022	0.082	0.105	*R*^2^ Δ = 0.042
	Social support			*F* Δ = 9.214**	0.113	0.021	*F* Δ = 9.197**
	PTSD				−0.162	0.002	
Psychological	Location	0.282	0.000	*R*^2^ Δ = 0.079	0.190	0.000	*R*^2^ Δ = 0.087
	Social support			*F* Δ = 35.381**	0.176	0.000	*F* Δ = 21.229**
	PTSD				−0.220	0.000	
Social relationship	Location	0.209	0.000	*R*^2^ Δ = 0.044	0.137	0.006	*R*^2^ Δ = 0.077
	Social support			*F* Δ = 18.729**	0.221	0.000	*F* Δ = 18.005**
	PTSD				−0.145	0.004	
Environmental	Location	0.288	0.000	*R*^2^ Δ = 0.083	0.155	0.001	*R*^2^ Δ = 0.170
	Social support			*F* Δ = 37.111**	0.222	0.000	*F* Δ = 46.365**
	PTSD				−0.330	0.000	

*R*^2^ Δ: *R* squared change; *F* Δ: *F* change; β: standardized regression coefficient; quality of life.

** *p* < 0.001.

## Discussion

The present study compared the quality of life in two communities in Nigeria with different exposures to conflict and investigated the inter-relationship between quality of life, PTSD, and social support. It revealed that the participants from the community directly exposed to the conflict had a poorer quality of life compared to those exposed indirectly. PTSD was observed to negatively influence all the subscales of quality of life, while only the family subscale of social support appeared to be consistently associated with improved quality of life.

The impact of direct exposure to trauma or conflict has far-reaching consequences on the quality of life of the affected people (Fatas et al., 2021; Yan et al., 2022). The present study has replicated this by showing the association between direct exposure to conflict and quality of life. Exposure to conflict has been associated with lower quality of life and has persisted even beyond the actual hostilities, possibly due to the destruction of health-supporting infrastructure such as employment, accommodation, security, and other socioeconomic post-conflict instability. For example, a study conducted among refugees in Nigerian revealed poor quality of life years after they had been moved away from the region of conflict (Akinyemi et al., 2012). This prolonged impact on the quality of life suggests the need to always look beyond the immediate mental and physical health consequences of exposure to conflict and that quality of life may be a better measure of post-conflict well-being. It also reiterates the need for relevant interventions in the broader context of lives, including but not limited to environment and social relationships, as all the areas of the quality of life were affected in the participants.

We also set out to examine the interaction of PTSD and social support with quality of life using a hierarchical model. Whilst the effect of location accounted for up to 8% of the variance for the quality of life, the addition of PTSD and social support further increased it to 17%, thus suggesting the huge role these variables play in determining the quality of life.

PTSD is an important mediator of poor quality of life as they have been negatively correlated (Matanov et al., 2013; Yang et al., 2021). A similar study had reported a significant difference in the occurrence of PTSD between the two groups of community (Edeh et al., 2023); the present one revealed a negative correlation with all the domains of quality of life, including physical, psychological, social relationships, and environmental. The higher rate of PTSD among those directly exposed could explain the difference in the quality of life observed between these two groups of individuals, as the former is an independent predictor of the latter (Matanov et al., 2013). PTSD severely impaired the quality of life in a longitudinal study where 854 refugees were followed for 8 years after the war in three Western European countries (Matanov et al., 2013). Also, a 6-month follow-up of admitted COVID-19 survivors revealed significant impairment of both mental and physical functions (Huang et al., 2022). PTSD, especially in its severe form, may affect the ability to work, perform day-to-day activities or relate to family and friends and may be responsible for the prolonged consequences of disaster despite the provision of rapid relief measures and relocation of the victims if untreated.

Conversely, the present study revealed that social support appeared to improve quality of life, as evidenced by their positive correlation. Its small explanation of the variance of quality of life in our study has been previously documented (Ke et al., 2010; Mizuno et al., 2009), thus suggesting the possibility that the relationship could be mostly indirect. For example, Sippel et al. (2015) suggested a mechanism by which social support protects against psychological stress and improves the quality of life, which includes building resilience in people exposed to a traumatic situation such as a disaster. Research has demonstrated that having attachment figures available can help regulate stress and arousal responses. It also promotes positive attitudes, such as increased self-confidence, avoidance of harmful coping mechanisms, and the development of effective problem-solving skills (Holahan et al., 1995). Whilst we had related traumatic disorders such as PTSD to quality of life, we hypothesized that social support improves the latter by protecting against the former and recommend further investigation on the dynamic of these relationships and how they can be used to develop preventive measures guidelines.

Family was the only social support domain that correlated with PTSD and quality of life in our study, as previously reported (Bos-Roubos et al., 2022). While the present study is cross-sectional and thus precludes us from inferring a causal relationship, it does propose a multifaceted correlation between PTSD, family support, and quality of life. A previous study has suggested an indirect relationship between family support and quality of life, indicating that the relationship between a psychiatric disorder and quality of life 6 months later was mediated by perceived emotional support from the family (García-Carmona et al., 2021). Further, a study conducted among injured service members in the United States found that individuals who received substantial family support reported lower levels of posttraumatic stress disorder (PTSD) when exposed to trauma (McCabe et al., 2019). The study also highlighted similar trends in terms of the quality of life of the affected individuals. Regardless of the direction of the relationship, these findings underscore the significance of interpersonal relationships and support for individuals who experience trauma. They also emphasize the need to address these topics in existing treatment and rehabilitation programs. Hence, it is germane to acknowledge and address the importance of family support as a crucial component in managing PTSD and improving the quality of life of those who have suffered from traumatic events.

Even though social support is believed to buffer the negative impact of stressful events, some researchers believe it is only beneficial when applied suitably (Lim and Zebrack, 2008; VonDras et al., 2008). The appropriateness of this variable depends on the cultural context, the type of life event, the individualities of the affected people, and the relationship between the provider and beneficiary. For example, while Asians are more likely to benefit from social networking, especially with close relatives, Caucasians would benefit from explicit social support, which includes event-specific advice (Taylor et al., 2007). Similar to the Asians (Taylor et al., 2007), our study reflects that family and friends are the major sources of support readily available in our culture.

Nonetheless, the finding of a strong relationship with the family domain reinforces the claim of an earlier manuscript, which described the impact of a close-knit family system in building resilience against mental exhaustion during COVID-19 and Ebola in West Africa (Raven et al., 2018) and suggested the likely appropriate area of therapeutic or preventive target in settings like ours (Raven et al., 2018). Moreover, in Nigerian communities, this type of family system has long served as a reliable source of social benefits, particularly with respect to sharing emotional resources. This system has proven to be a cost-effective means of coping during times of crisis. The study further reiterates the importance of a strong social support network in bolstering individuals during times of adversity.

### Limitation

We advise a cautious interpretation of the present study due to some methodological limitations. These include the generalizability of the result to other parts of the country with cultural differences and the lack of causal relationships between the outcome and the independent variables due to its cross-sectional nature. It is worth noting that utilizing differences in location to deduce the impact of direct and indirect exposure to conflict is a somewhat theoretical approach that requires careful interpretation. It is necessary to exercise caution when drawing conclusions from this methodology as other confounding variables, such as other life events which were not controlled for, could potentially account for the observed differences. Therefore, it is important to consider additional explanations and to conduct further analysis, such as incorporating all potential confounders to gain a more comprehensive understanding of the underlying dynamics at play. In addition, the manner in which the previous history of a mental illness was screened was subjective; it would have been more valuable to apply a standardized scale to rule out the presence of pre-existing psychopathology. Further, the use of the CIDI, social support scale, and WHOQOL instruments, which are yet to be validated in Nigeria, may introduce cross-cultural invalidity; however, some measures were put in place to overcome this drawback such as the use of interviewers who were mental health professionals and who could speak the Tiv and English languages fluently. Finally, it would have been valuable to explore the impact of PTSD and quality of life in the various age group because of the possible impact of age in confounding the results found in this study; nonetheless, such analysis is beyond the scope of this study.

### Implication on policy and practice

The present study brings to light some issues that we believe could impact policies and practices. In many African communities, particularly in Nigeria, where political instability and constant conflicts are prevalent, affected individuals tend to overlook the complications and the future implications of trauma. People in this part of the world often struggle to continue with their daily activities, acting as though nothing has happened, and resort to maladaptive coping mechanisms such as drug abuse and aggression toward significant others in their lives. Trauma can make individuals believe that they are worthless, undeserving of happiness, and incapable. They may view life as meaningless, believing they do not deserve happiness or will never achieve their goals. They may also lose faith in others and the world around them and continue to struggle to envision a future for themselves. As a result, most of them do not seek help even when it is apparent that they need it.

Our research highlights the long-term impact on the quality of life. It thus suggests the need for policymakers and healthcare planners to consider the broader context of individuals’ lives, including their social environment and relationships, and to always look beyond the immediate mental and physical health consequences of exposure to conflict. Quality of life may be a better measure of post-conflict well-being. The study also emphasizes the need for relevant interventions to address the various areas of quality of life affected by the participants.

Other healthcare professionals should pay attention to indicators of the covert mental consequences of untreated PTSD, such as reduced productivity, psychosomatic symptoms, poor response to medical treatment, and overall poor quality of life.

We encourage more studies from settings like Nigeria, where there is unrelenting conflict, terrorism and political instability, to aid in the planning and implementing short- and long-term relief and healthcare services for people affected by conflicts.

## Conclusions

To sum up, our research has shown that being directly exposed to crises can negatively impact one’s quality of life. Specifically, it increases the risk of experiencing psychological and physical health issues, as well as environmental damage. PTSD was observed to play a significant role in the quality of life of the people exposed to crises, while only the family domain of social support was protective of both PTSD and quality of life. Although further research is necessary to support our assertion, the current findings indicate that implementing preventive initiatives to enhance the quality of life should be a policy focus in areas directly impacted by traumatic conflict. This should include prioritizing strengthening family ties in affected regions, as it has been proven to be a valuable resource during times of crisis and can provide maximum benefit. Further, appropriate therapeutic intervention should be provided to individuals with PTSD to aid their quality of life.
